# The Effectiveness of GECB Pastille in Reducing Complications of Dry Socket Syndrome

**DOI:** 10.1155/2012/587461

**Published:** 2012-04-22

**Authors:** Abbas Haghighat, Rahim Bahri Najafi, Mostafa Bazvand, Hamid Badrian, Navid Khalighinejad, Hossein Goroohi

**Affiliations:** ^1^Department of Oral & Maxillofacial Surgery and Torabinezhad Center Research, Dental School, University of Medical Sciences, Isfahan, Iran; ^2^Department of Pharmaceutics, Pharmacy School, University of Medical Sciences, Isfahan, Iran; ^3^Dentistry School, University of Medical Sciences, Isfahan, Iran

## Abstract

*Background and Purpose*. Dry socket syndrome is one of the most irritating complications after tooth extraction. This study aims to investigate the efficacy of pastille GECB compared to ZOE. *Materials and Methods*. 30 patients with dry socket syndrome were selected and divided into two groups. GECB pastille was produced with 3% Guaiacol, 3% Eugenol 1.6% Chlorobutanol, sized 3 × 7 × 10 mm. GECB was applied in one group, and ZOE was used for the other group. Duration of pain after treatment and painkiller intake values were recorded within 20 days. The data were analyzed with independent samples *t*-test, Mann-Whitney, and Chi-Square tests. *Results*. Pain persisted for 45.53 ± 33.34 minutes in patients treated with ZOE and 19.87 ± 21.80 minutes in those treated with GECB (*P* = 0.19). Patients in the ZOE group reported more painkiller intake within 20 days (*P* = 0.031). *Conclusion*. GECB showed more significant efficacy in reducing complications after tooth extraction.

## 1. Introduction

Pain, bleeding, infection, paresthesia, temporomandibular disorders, abscess formation, and dry socket have been known as common complications following extraction of impacted wisdom teeth [1, 2]. Dry socket syndrome was introduced for the first time by Crawford. This syndrome is characterized by severe pain in the extraction and periextraction site accompanying by blood clot lysis and halitosis [3]. In Nusair and Abu Younis study, 7.9% was the highest rate of dry socket syndrome that occurred in the range 18–33 years old [4]. The occurrence of this syndrome after extraction of other teeth than third molar is about 2% while the incidence of this syndrome after third molar extraction is 20% [5]. Also in Haghighat study, it was concluded that there is a significant correlation between the use of oral contraceptive pills, traumatic extraction, and smoking cigarette with occurrence of postoperative dry socket syndrome [6]. 

Until now, different preventive and therapeutic methods have been proposed based on multifactorial and complex etiology of dry socket syndrome. Some studies focused on antibiotics and antibacterial agents as an effective way in the management of dry socket syndrome. In Swanson [1] and Akota et al. [2] studies, the use of antibiotics was shown effective in the management of the syndrome. Beside antibiotics, other studies concentrated on the effect of 12% chlorhexidine mouthwash, and it was declared that this agent significantly reduces the dry socket syndrome complications compared to control group [7]. Cai and Lu investigated the efficacy of Gelatamp colloidal silver gelatin sponge in prevention of postextraction complications and concluded that Gelatamp was effective in prevention of dry socket syndrome [8]. In Keesling and Keats study, zinc oxide eugenol ointment was compared with placebo, regarding the dry socket prevention, and it was shown that ZOE is more effective than placebo [9].

From different suggested methods, Guaiacol, Eugenol, Chlorobutanol, and Balsam peru mixture (G.E.C.B) is a new material, introduced by Sultan company in the name of Dentalone. This mixture is a paste, consists of 3% guaiacol, 3% Eugenol, and 1.6% Chlorbutanol as an effective agent and Balsam peru as a base. According to therapeutic and alleviative effects of its ingredients, this paste was released by Sultan chemists in the name of Dry socket paste.

Dry socket syndrome is one of the most painful complications following impacted third molar extraction, and it can affect both patients and clinicians emotionally and financially. As there are no definite explanations for the occurrence of this syndrome and prevention of this syndrome is an important duty for clinicians, the aim of this study is to investigate the efficacy of the new form of GECB drug as pastille in management of dry socket syndrome-related complications.

## 2. Methods and Materials

### 2.1. Patient Selection

In this randomized clinical trial, 30 patients aged from 18 to 40 who referred to maxillofacial surgery department of Isfahan University of medical sciences and diagnosed with dry socket syndrome after impacted lower third molar extraction were selected. These patients consisted of 7.5% of the whole patients. Patients with allergy to either of the dressing materials used in the study, infection in the site of tooth extraction and those who refused to participate in the study, were excluded. Patients who met the inclusion criteria (pain in the lower arch following tooth extraction which can refer to ears, exposure of alveolar bone, and empty alveolar socket) were randomly divided into two groups. Fifteen patients (11 males and 4 females) undergone treatment with ZOE, and fifteen patients (7 males and 8 females) were treated with pastille GECB. An informed consent was signed by patients, and the study has been ethically approved by Isfahan dental school (no. 390053).

### 2.2. GECB Pastille Preparation

GECB pastille was prepared according to official formula which has been released by Sultan Company and approved by FDA. 3% eugenol (Merck), 3% Guaiacol (Merck), and 1.6% chlorobutanol are the effective ingredients in the pastille. All mentioned ingredients were inserted in the gelatin pastille formulation containing gelatin, glycerin, and sugar. After mixing all together, the mixture was poured in 3 × 7 × 10 mm mold. Molds were allowed to get cool, and then flexible pastilles were ready to use.

### 2.3. Medications Application

First, patients' pain was assessed by visual analog scale (VAS). In the case group, GECB pastille and in control group ZOE dressing was used. After pastille and ZOE placement, the elapsed time for the patient's pain to significantly relieve was recorded. After the treatment, ibuprofen pills were prescribed as needed (PRN). Patients were followed for 20 days, and the pain severity was assessed by VAS, and the number of consumed pills was recorded after this period. Data were analyzed by Mann-withney and independent sample *t*-test using SPSS software versus 15 (*α* = 0.05).

## 3. Results

In the present study, 30 patients with mean age of 29.87 ± 6.00 were assessed. The mean age of patients was 28.80 ± 5.52 and 30.93 ± 6.64 in ZOE and GECB group, respectively. The mean age of patients showed no significant difference between two groups (*P* = 0.339).

73% and 47% of the patients were male in ZOE and GECB groups, respectively. Also there was no significant difference between two groups regarding their gender (*P* = 0.136) as illustrated in Table 1.

In the present study, the severity of pain before any treatment was also assessed by VAS scale in each patient. The mean score was 8.33 ± 1.05 and 8.27 ± 0.7 in ZOE and GECB groups which showed no significant difference (*P* = 0.839).

Independent sample *t*-test revealed that there is a significant difference between two groups in terms of the time elapsed for the pain to significantly reveal after the treatments (*P* = 0.019). In the ZOE and GECB groups, the pain reduced after 45.53 ± 33.34 and 19.87 ± 21.80 minutes, respectively.

Patients, who received ZOE and GECB treatments, averagely used 0.53 ± 0.74 and 0.07 ± 0.26 400 mg ibuprofen pills during the follow-up period. Mann-Whitney test showed that there was a significant difference in the number of consumed pills between the two groups (Table 2).

## 4. Discussion

Despite the importance of the dry socket syndrome, many clinicians are not willing to discuss about a definite treatment approach toward this syndrome, as the etiology, and the treatment of this syndrome is highly controversial [10]. Until today, symptomatic treatments are the most common way for pain alleviation. Considering the fact that the pain in dry socket syndrome will last for 10–15 days regardless of the treatment method, all the suggested treatment has focused on the pain reduction [10, 11]. Some researchers believed that the use of dressing pastes, not only delivers the effective material to the involved site, but also acts as a barrier for the socket. It should be emphasized that dressing materials should not act as a hindrance for wound-healing procedure and the dressing must be replaced with socket rinsing [12, 13].

In the present study, the mean age of patients showed no significant difference between the two groups. It is an important factor to consider, as some studies indicate that the incidence of dry socket syndrome increases with age [14]. So to negate the effects of this factor as confounding variable, the patients were monitored regarding their age.

Moreover, the primary pain before treatment was not significantly different between the two groups which cannot have any confounding effect on the results of the study.

The pain rate in the GECB group was significantly lower, regarding its duration and the number of required pain killers. But it should be added that ZOE dressing is an acceptable treatment [15].

Guaiacol is an eugenol-like phenolic combination [16]. This material, for the first time, was used as cement in dentistry and also used as an adjunctive treatment for pains with pulp origin [17, 18]. Also like other phenolic combinations, guaiacol can induce cellular proliferation, and this feature limited the use of this material [19]. It is believed that the alleviative effect of ZOE is due to its eugenol. As Eugenol is present also in GECB pastille and the effect of GECB is superior to ZOE, it can be concluded that the alleviative effect of Guaiacol is more than that of Eugenol [20].

One of the major advantages of GECB over traditional ZEO dressing is its convenience. As the treatment of dry socket syndrome by ZOE needs dressing replacement each 3 days for 1 week, some researchers consider it as an impediment in the treatment procedure [21]. In the present study, GECB pastille which is a mixture of medicament in gelatin and sugar was used. This pastille is a solid, transparent material with pleasant taste, and it does not need preparation before its use. This significantly saves the patients' time and is more convenient for them. As the entire ingredients in the pastille have been approved by F.D.A, the mentioned pastille also can be approved and released in the market with different concentrations.

The comparison of the present study with other studies is challenging as the pastille form of this drug was used for the first time in the treatment of dry socket syndrome. Despite the conclusive results of the present study, further studies with bigger sample sizes and different concentrations of the medicament should be conducted.

## 5. Conclusion

It can be concluded that there is a significant difference in the capacity of two different methods in pain alleviation. It seems that this effect is mainly due to use of Guaiacol. Also because of GECB pastille convenience and its one session use with less chair time, compared to ZOE, it can be an effective and reliable alternative for traditional ZOE method.

## Figures and Tables

**Table 1:** Comparison of age and VAS-scale of the patients between the groups.

Criteria	Group	*N*	Mean	Std. deviation	Std. error mean
Age	ZOE	15	28.80	5.51	1.42
	GECB	15	30.93	6.46	1.66
VAS-scale	ZOE	15	8.33	1.04	0.27
	GECB	15	8.27	0.70	0.18

**Table 2:** Healing time and ibuprofen intake compared between the two groups.

Criteria	Group	*N*	Mean	Std. deviation	Std. error mean
Healing-time	ZOE	15	45.53 min	33.33	8.60
	GECB	15	9.87 min	7.62	1.96
Ibuprofen	ZOE	15	212 mg	0.74	0.19
	GECB	15	32 mg	0.25	0.06
